# Characterization of a Bacteriophage GEC_vB_Bfr_UZM3 Active against *Bacteroides fragilis*

**DOI:** 10.3390/v15051042

**Published:** 2023-04-25

**Authors:** Nata Bakuradze, Maia Merabishvili, Ia Kusradze, Pieter-Jan Ceyssens, Jolien Onsea, Willem-Jan Metsemakers, Nino Grdzelishvili, Guliko Natroshvili, Tamar Tatrishvili, Davit Lazvliashvili, Nunu Mitskevich, Jean-Paul Pirnay, Nina Chanishvili

**Affiliations:** 1 Laboratory of Microbial Biotechnology, Eliava Institute of Bacteriophages, Microbiology and Virology, Tbilisi 0160, Georgia; 2 Department of Biology, Faculty of Exact and Natural Sciences, Javakhishvili Tbilisi State University, Tbilisi 0179, Georgia; 3 AIETI Medical School, Davit Tvildiani Medical University, Tbilisi 0159, Georgia; 4 Laboratory for Molecular and Cellular Technology, Queen Astrid Military Hospital, 1120 Brussels, Belgium; 5 Laboratory of General Microbiology, Eliava Institute of Bacteriophages, Microbiology and Virology, Tbilisi 0160, Georgia; 6 Faculty of Medicine, European University, Tbilisi 0141, Georgia; 7 Unit of Human Bacterial Diseases, Sciensano, 1180 Brussels, Belgium; 8 Department of Trauma Surgery, University Hospitals Leuven, 3000 Leuven, Belgium; 9 Department of Development and Regeneration, KU Leuven, 3000 Leuven, Belgium; 10 Faculty of Natural Science and Medicine, Ilia State University, Tbilisi 0162, Georgia

**Keywords:** *Bacteroides fragilis*, phage therapy, bacteriophage

## Abstract

*Bacteroides fragilis* is a commensal gut bacterium that is associated with a number of blood and tissue infections. It has not yet been recognized as one of the drug-resistant human pathogens, but cases of the refractory infections, caused by strains that are not susceptible to the common antibiotic regimes established for *B. fragilis,* have been more frequently reported. Bacteriophages (phages) were found to be a successful antibacterial alternative to antibiotic therapy in many cases of multidrug-resistant (MDR) bacterial infections. We have characterized the bacteriophage GEC_vB_Bfr_UZM3 (UZM3), which was used for the treatment of a patient with a chronic osteomyelitis caused by a *B. fragilis* mixed infection. Studied biological and morphological properties of UZM3 showed that it seems to represent a strictly lytic phage belonging to a siphovirus morphotype. It is characterized by high stability at body temperature and in pH environments for about 6 h. Whole genome sequencing analysis of the phage UZM3 showed that it does not harbor any known virulence genes and can be considered as a potential therapeutic phage to be used against *B. fragilis* infections.

## 1. Introduction

Treatment of bacterial infections with bacteriophages has been practiced in Georgia and a few other Eastern European countries, such as Poland and Ukraine, since the beginning of 20th century, even before the issues with antibiotic resistance were on the horizon [1]. Since the worldwide emergence of MDR pathogens [2], the experience regarding phage therapy has been shared with the rest of the world [3]. *Bacteroides fragilis* is a Gram-negative, anaerobic, rod-shaped bacterium. Naturally, *B. fragilis* is found in the normal gut microbiome of humans and some other mammals. Colonizing the mucosal lining as a commensal bacterium, it is involved in a number of physiological processes, such as food digestion and maturation of the immune system [4]. However, it is an opportunistic agent which produces mixed anaerobic infections involving other residents of the intestinal tract after their transition to other tissues [4]. Lately, the increased occurrence of the resistant strains leads to occasional failures in treatment of the infections caused by *B. fragilis* [5]. The first case, reported in 2011, occurred in a U.S. Army soldier with MDR *B. fragilis* isolated from blood and tissue following an injury sustained in Afghanistan [6]. In 2013, an MDR *B. fragilis* strain was isolated from the bloodstream and intra-abdominal abscesses of a patient who had previously received treatment in India. This strain appeared to be resistant to both carbapenems and metronidazole [7]. Merchan et al. [8] described a case of *B. fragilis* bacteremia associated with paraspinal and psoas pelvic muscle abscesses in the United States. Resistance to beta-lactam/beta-lactamase inhibitors, carbapenems, and metronidazole was determined. Drug-resistant strains of *B. fragilis* have been reported in Denmark [9], Hungary [10], and China [11], and a fatal case was reported in Japan as well [12].

*B. fragilis* is categorized into two subgroups: non-enterotoxigenic *B. fragilis* (NTBF) and enterotoxigenic *B. fragilis* (ETBF). ETBF strains contain *bft* gene coding for enterotoxin, which is a metaloprotease that cleaves intercellular e-cadherin protein [13]. This cleavage process is thought to be involved in pathogenesis of diarrhea as well as initiation of cancer [14]. Therefore, ETBF is likely to be a contributing factor in carcinogenesis [15,16]. In developing counties, ETBF is an emerging pathogen which is associated with diarrhea in children (age, 1–5 years) and travelers. In children, infection with ETBF leads to mild secretory diarrhea. The other diseases caused by ETBF as well as NTBF include: extra-intestinal infections, abdominal pain, tenesmus, inflammatory and antibiotic associated diarrhea, and chronic inflammation that can lead to colon cancer [16,17]. The worldwide emergence of antibiotic resistant strains of *B. fragilis* leads us to consider the possible use of phage therapy for the treatment and prophylaxis of the infectious diseases it causes. The only application of phages active on *B. fragilis* studied so far is the detection and monitoring of the fecal contamination of water [18]. However, some novel lytic bacteriophages applicable for phage therapy have been described recently [19].

We have studied a bacteriophage GEC_vB_Bfr_UZM3 active on *B. fragilis*, which was applied to treat the chronic osteomyelitis caused by the mixed infection of *B. fragilis*, *Staphylococcus aureus,* and *Pseudomonas aeruginosa* strains in an adult patient in 2019. UZM3 was administered in combination with ISP and 14-1, which are well-studied phages and have been used for the treatment of *S. aureus* and *P. aeruginosa* infections, respectively [20,21]. The phage mixture with the titer of 10^7^ PFU/mL was applied locally, on a daily basis, for ten days [22]. No side effects were detected during the phage therapy. Eradication of the targeted bacteria could not be determined as the patient died during the follow-up period due to comorbidities. Ethical approval was obtained from the Ethics Committee Research UZ/KU Leuven. Informed consent was obtained from the patient.

## 2. Materials and Methods

### 2.1. Isolation and Propagation of Bacteriophage

*B. fragilis* phage UZM3 was isolated from a wastewater sample of the University hospital of Ghent (Belgium) in 2015 and was pre-adapted on the clinical isolate of *B. fragilis* UZ-10 in 2019.

UZ-10 originated from a patient with chronic osteomyelitis of the left sacrum at the University Hospitals Leuven (Belgium).

The preadaptation process of the phage was performed in BHI broth culture by incubating UZ-10 clinical strain and the phage at multiplicity of infection (MOI): 0.01 with the final concentration of bacteria ~1–3 × 10^8^ CFU/mL and phage ~1–3 × 10^6^ PFU/mL for 24–48 h, at 37 °C anaerobically in 10% CO_2_ atmosphere.

Following the incubation, the lysate was centrifuged (20 min at 6000× *g*), and the supernatant was filtered through a 0.22 µm pore filter. Further, the filtrate was tested on the presence of phage virions using the double layer agar method (DLA) [23] to reveal the lytic plaques. Next, the dilutions of the filtrate (10^−2^–10^−8^) were mixed with the 100 mL of UZ-10 fresh culture and 2–3 mL of BHI overlay medium (0.6% *v*/*w* of agar) and, after gentle mixing, poured on the solid BHI medium. The plates were incubated at 37 °C in a 10% CO_2_ atmosphere. Afterwards, the produced plaques were selected, cut out, and incubated in broth with the bacteria at 37 °C for 2 h anaerobically in a 10% CO_2_ atmosphere.

The procedure was repeated 4 times. The phage UZM3 propagated on the clinical strain UZ-10 was sequenced in order to reveal the virulence factor coding genes in the genome of the phage.

For the experiments of biological characterization, phage UZM3 was propagated on the *B. fragilis* A7 strain, which was found to be a better host for the replication of UZM3. A7 was easier to culture than the clinical strain UZ-10 and gave a higher yield of phages in the DLA. The A7 strain was isolated from a stool sample in Georgia. It was also the host for the other two *B. fragilis* phages (VA7 and MTK) found in the phage collection of the Eliava Institute. A conventional PCR technique was performed for the detection of enterotoxin coding genes and the strain proved to be negative for the presence of *bft* [23,24].

### 2.2. Morphological Characterization

Transmission electron microscopy (TEM) was used for the visualization of the phage particles. For that, 10 μL of a phage suspension with the titer of 3 × 10^10^ PFU/mL was spotted onto carbon-coated grids and stained with 1% uranyl acetate. The negatively stained grids were observed using the Jeol 100 -SX transmission electron microscope at 80 kV.

### 2.3. Host-Range Evaluation

The host range of the UZM3 propagated on *B. fragilis* host A7, along with the other two phages GEC_vB_Bfr_VA7 (VA7) and GEC_vB_Bfr_MTK (MTK) [23], was evaluated against fifteen isolates of ETBF as well as non-ETBF *B. fragilis* from the Eliava Institute bacterial culture collection. All 15 isolates, including A7, were isolated in Georgia from the stool samples of patients who had non-*B. fragilis*-related pathologies. The precise identification of the bacterial isolates was performed by using MALDI–TOF mass spectrometry [23]. We have also tested the spectrum of activity of the UZM3, which was adapted on the clinical strain (UZ-10). Ten microliters of the phage suspensions of *B. fragilis* isolates with the titer of 1 × 10^8^, 10^7^,10^6^ PFU/mL were spotted on the lawns of BHI solid agar medium. One hundred microliters of 1–3 × 10^8^ CFU/mL of the overnight bacterial cultures grown in BHI broth with supplements (Hemin, Vit K) and mixed with 2–3 mL of BHI overlay medium were used to make bacterial lawns on the petri dishes with the DLA method. The results were considered positive when the lysis zones or plaques were formed in more than one dilution of the phage solution as a sign of its bactericidal activity [25]. We have assessed the lytic effects by numbering them from 4 to 1, indicating strongest to weakest (depicted in the Supplementary Material Table S1).

### 2.4. Temperature and pH Stability Assay

UZM3 phage solution with the titer of 3–5 × 10^8^ PFU/mL was diluted in 0.9% NaCl. The diluted phage was incubated at different temperature conditions (4 °C, 25 °C, 37 °C, 40 °C, 55 °C, 60 °C, 70 °C) and was checked at various time points within 6 h to ensure the stability of the phage concentration. To study the stability of UZM3 phage in acidic and alkaline conditions (pH 3, 5, 7, 9, 11), the phage suspension at concentration of 2 × 10^6^ PFU/mL diluted in SM buffer (200 mM NaCl, 10 mM MgSO_4_, 50 mM Tris-HCl, pH 7, 5) was incubated at room temperature and checked every 2 h within 6 h. For the evaluation of the results of the stability at various temperature, as well as pH, the spot-test assay was used [26]. Every 2 h, the phage concentrates incubated at the specific temperature or pH points were titrated and 10 μL from each dilution was distributed as a drop on the bacterial lawn prepared with the DLA method. After the drops of phage dilutions were air-dried, the plates were incubated for 24 h at 37 °C in a 10% CO_2_ atmosphere.

### 2.5. One-Step Growth Curve

We studied the one-step growth curve of the UZM3 phage to determine the latent period of the virus and the burst size of the infected host cells. The experiment was accomplished according to Kropinski [26]. Bacterial strain *B. fragilis* A7 was grown in 5 mL of BHI broth for 18 h at 37 °C in a 10% CO _2_ atmosphere. At the exponential growth phase, the *B. fragilis* A7 culture was diluted in BHI broth enriched with 1 mM of CaCl_2_ in order to reach the final concentration at 1 × 10^7^ CFU/mL. UZM3 lysate was added to the bacterial culture in order to reach the MOI of 0.01 (i.e., the final phage titer was equal to 1 × 10^5^ PFU/mL). The phage–bacterial mixture was incubated in a water bath at 37 °C for 6 min in order to achieve phage-host cell adsorption, 0.1 mL of this mixture was further ten-fold diluted up to 1 × 10^1^ PFU/mL. At the same time, 0.01 µL of CHCl_3_ was added to 1 mL of the 1 × 10^2^ PFU/mL phage dilution to serve as an adsorption control, which was stored on ice till the end of the experiment. We incubated the 1 × 10^3^, 1 × 10^2^, and 1 × 10^1^ PFU/mL concentrations of the phage with the bacterial culture at 37 °C in a water bath for 60 min. At five-minute intervals, 0.1 mL of each dilution was mixed with 0.1 mL of *B. fragilis* A7 bacterial culture and BHI overlay agar, and was applied on a 1.5% BHI solid agar medium; 0.1 mL of the adsorption control was also plated using the same DLA method. The plates were incubated for 24 h at 37 °C in a 10% CO_2_ atmosphere. The number of infected cells and the burst size were determined as a ratio of the average number of viral particles after the eclipse phase and the average number of virions during the latent phase [27].

### 2.6. Whole Genome Sequencing and Analysis

Phage DNA was extracted by using the MagCore^®^ Viral Nucleic Acid Kit (Code 201, Atrida, Amersfoort, Netherlands NL) and genome was sequenced using the Illumina MiSeq™ Platform (Illumina, San Diego, CA, USA) at Sciensano (Brussels, Belgium). Short-read sequencing libraries were prepared with an Illumina Nextera XT DNA Library Preparation Kit and sequenced on an Illumina MiSeq instrument with a 250 bp paired-end protocol (MiSeq v3 chemistry), according to the manufacturer’s instructions. Trimming of the short reads was performed with Trimmomatic (version 0.32) [28]. First, the Illuminaclip option was used to remove the Nextera adapter sequences. Then, a sliding window approach of four bases and trimming when the Phred score dropped below 30 was employed. Lastly, the leading and trailing bases of a read were removed when the Phred score dropped below 3. All reads that were smaller than 50 bp were removed. De novo assembling was performed by using spades (v. 3.15.3). Assembled data with the coverage 290× was proceeded for annotation. For the manual annotation, the Artemis [29] annotation tool was used. The putative open reading frames (ORFs) were predicted by using GenemarkS (v. 4.28) [30]. Functions of the ORFs were analyzed by PHROGs version 4 and HHpred software [31,32]. The genome map was generated with Geneious software [33]. Comparative genomics was performed by using the Easyfig (v.3.4) tool. The viral proteomic tree was constructed by VIPtree (v.2.2.5) [34,35]. Genome sequence identity was calculated by VIRIDIC (v. 1.1) [36]. The prediction of tRNAs was performed by using tRNAscan-SE (v.1.3.1) software [37]. The sequence has been uploaded to GenBank with the accession number: OQ116603.

## 3. Results

**Figure 1:**
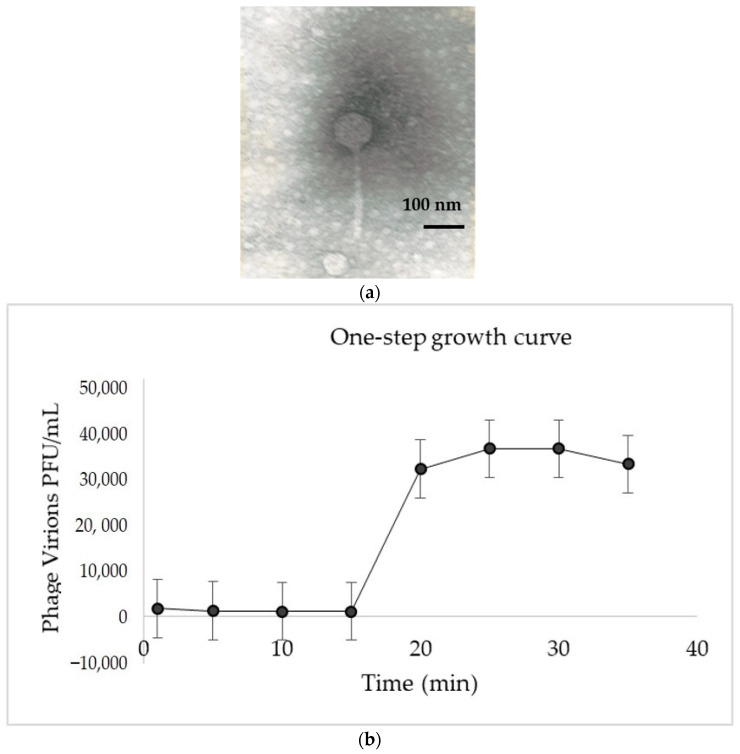
Results of morphological and biological characterization of UZM3. (**a**) TEM image of UZM3 virion (Magnification × 230,000); (**b**) One-step growth curve of UZM3. The results represent the mean of 3 replicates.

We have characterized phage UZM3 that was used for the treatment of the polymicrobial osteomyelitis, including the *B. fragilis* strain. Transmission electron microscopy of the phage revealed a typical morphology of a siphovirus (morhotype) with the icosahedral head diameter of about 65 nm and the non-contractile tail of 139 nm length (Figure 1a.). The one-step growth curve of the virus shows that the latent period of UZM3 lasts about 10 min and the burst size is about 30 virions per cell (Figure 1b).

**Figure 2:**
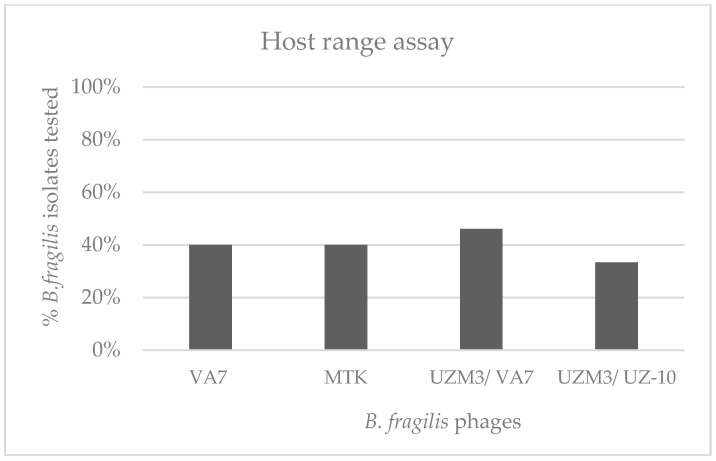
Activity spectrum of the phages MTK, VA7, and UZM (propagated on UZ-10 and A7 strains), as well as phages against the 15 clinical isolates of *B. fragilis*.

The host range evaluation performed with the spot-test assay showed that UZM3, which was propagated on the A7 (UZM3/A7), had a lytic activity on the nine bacterial isolates, showing a slightly wider spectrum of activity than the other phages tested. Interestingly, when propagated on the clinical strain (UZM3/UZ-10), the host range was limited to five bacterial isolates. (Figure 2).

**Figure 3:**
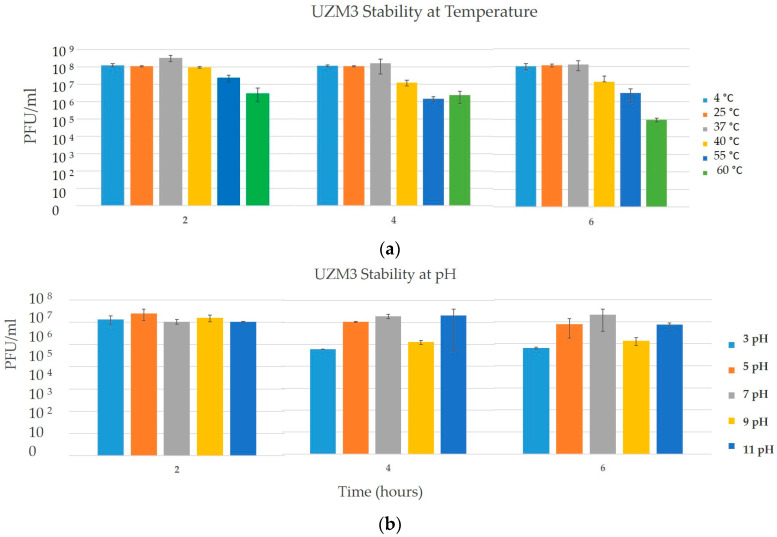
Results of UZM3 stability at various temperatures for 24 h (**a**) and pH (**b**) points for 6 h. The results represent the mean of 3 replicates.

The phage UZM3 maintained its stability at 4 °C, 25 °C and 37 °C for the 6 h. At 40 °C there was a decrease in viral activity by one logarithmic (log) phase after 4 h. At higher temperature points (55 °C, 60 °C), the active viral numbers dropped by one to two log phages after 2 h, but did not go below 10^6^ PFU/mLwithin 6 h of observation (Figure 3a). Based on the 6 h study on pH stability the phage, UZM3 appears to withstand both the acidic as well as the alkaline (3–11 pH) environments for 4–6 h with only the loss of the concentration by one or two logarithmic units (Figure 3b).

**Figure 4:**
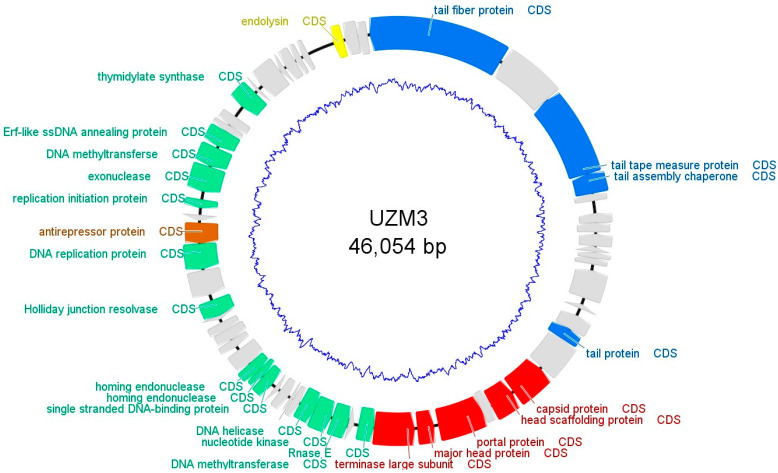
Phage UZM3 genome map. Gene functions are color-coded as follows: regulatory modules, tail modules, the DNA packaging and head module; hypothetical protein encoding genes are depicted as orange, blue, red and grey arcs, respectively.

The sequence analysis revealed that phage UZM3 has a small genome of 46,054 bp with the GC content of 38.89%. A total of 61 ORF were identified that correspond to 41,772 bp gene coding region. Among the 61 ORFs, only 25 ORFs were functionally annotated (Supplementary Material Table S2). ORFs with the predicted functions were grouped as follows: (1) DNA packaging and head morphogenesis module (5 ORFs); (2) tail morphogenesis module (4 ORFs); (3) cell lysis gene (1 ORF); (4) DNA, RNA metabolism (13 ORFs) and regulatory gene with one ORF (Figure 4).

**Figure 5:**
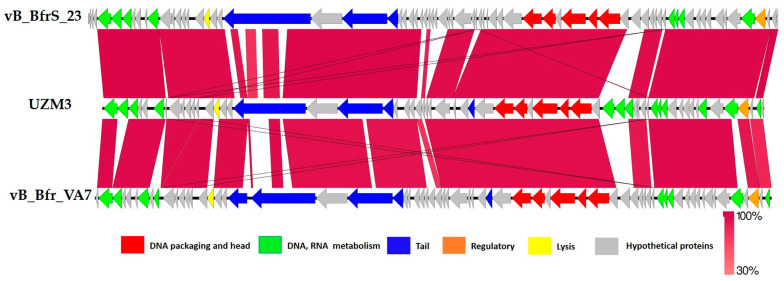
Comparison and annotation of phage GEC_vB_Bfr_UZM3, vB_BfrS_23, and GEC_vB_Bfr_VA7 genomes. Gene functions are color-coded and detailed as follows: regulation, brown; tail, blue; DNA packaging and head, red; lysis, yellow; DNA and RNA metabolism, green; (hypothetical proteins encoding genes are depicted grey).

A Blastn similarity search revealed that UZM3 was the most similar to the virulent phage vB_BfrS_23 (89% query coverage) (GenBank accession number: MT630433) followed by the phage GEC_vB_Bfr_VA7 (83% query coverage), (GenBank accession number: MW916539). The study showed that an average nucleotide identity (ANI) for UZM3 and the most similar phages are less than 95% (84% for vB_BfrS_23 and 79% for GEC_vB_Bfr_VA7). The results indicate that phage UZM3 belongs to a new species [38]. The linear genome comparison of the UZM3, vB_BfrS_23, GEC_vB_Bfr_VA7, is illustrated in Figure 5. A viral proteomic tree of UZM3, and other similar phage genomes present in the NCBI database, revealed homology with other *B. fragilis* phages only (Figure 5).

**Figure 6:**
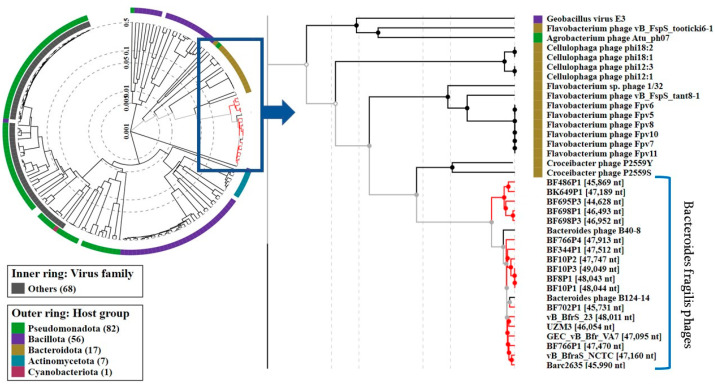
Evolutionary relationships between UZM3 phage and other viruses related to *B. fragilis*. Viral proteomic tree constructed by VIPtree.

Using the ViPTree, a comparative total proteome comparison was carried out in order to obtain a better idea of the evolutionary relationships between UZM3 and other viruses related to *B. fragilis*. All the *B. fragilis* phages given in Figure 6 are of *Caudoviricetes* class and we have determined that UZM3 is the closest to vB_BfrS_23.

## 4. Discussion

Phage therapy has become an emerging branch of personalized medicine due to the current prevalence of MDR bacterial infections [39,40]. The need for application of new bacteriophages has been increasing on an everyday basis. However, selection of phages for highly epidemic antibiotic-resistant species such as *Escherichia coli* or *P. aeruginosa* is easier to accomplish as they are widely distributed in the environment and can be easily isolated for therapeutic purposes. Subsequently, facilities performing phage therapy own a broad collection of viruses specific to the aforementioned pathogenic bacteria. On the other hand, pathogens such as *B. fragilis* do not belong to antibiotic-resistant organisms that are of primary concern. However, once in a while, they cause infections that are impossible to treat with the existing antibacterial solutions [11,39]. At the same time, isolation of bacteriophages specific for *B. fragilis* remains to be one of the challenging tasks resulting from the limited number of the studied phages.

It should be noted that, so far, bacteriophages active on *Bacteroides* species have been proposed as a possible indicator for fecal contamination due to their specific association with the excremental material and exceptional resistance to environmental conditions. Two groups of *B. fragilis* phages are used as biological agents for the assessment of water fecal contamination [40]. One is a restricted group of phages that specifically uses *B. fragilis* strain HSP40 as a host. The number of these phages in the sewage appear to be relatively low, and they are almost absent in some geographical areas. The *B. fragilis* HSP40 phages are siphoviruses (morphotypes), with the flexible non-contractile tails, double stranded DNA, and capsids with a diameter of up to 60 nm. The second group of *Bacteroides* phages have *B. fragilis* strain RYC2056 as a host [41]. This group includes a substantially wider spectrum of phages belonging to different morphotypes and harboring double stranded DNA. They are found in the feces of humans and many other animals. The number of these phages in the sewage are, as a rule, substantially higher than those of *B. fragilis* HSP40 phages [18]. However, as mentioned above, these phages are not always suitable for tracking the fecal pollution in the different geographical zones. For example, the phages successfully used for identification of fecal contamination in the UK and Spain were much less efficient for the US environment [42].

The aim of the present research was to study and characterize *B. fragilis* bacteriophages from the perspective of phage therapy. Using the adaptation method to the patient’s bacterial strain UZ-10, we have acquired *B. fragilis* phage UZM3. Biological, morphological, and genetic characterization of the virus revealed the siphovirus morphotype, which is characterized with the strong lytic activity against the clinical strain. The genome of UZM3 does not contain most of the genes required for the lysogenic properties of the phage. However, one anti-repressor gene, which was assigned by Phrogs system as the regulatory gene, is known to be involved in the lysogenic life cycle of the temperate phages. The function of the anti-repressor protein is mainly found to be associated with the switching of the life cycle of the temperate phage from a lysogenic to a lytic state [43]. However, as no other primary genes for the lysogenic cycle, such as integrases or excisionases [44], were identified, we assume UZM3 to be a lytic phage. Within the genome of UZM3, no known virulent genes for antibiotic resistance or toxins were identified. That is another advantage of the phage being used for the therapy of the bacterial infections, as the chance of transferring pathogenic properties to the target bacteria is low [44].

The host range assay showed that the spectrum of activity of UZM3 varied depending on the host it was propagated on. The study revealed that the UZM3 variant replicated on A7 became more active on Georgian bacterial isolates but lost its lytic effect on the clinical strain UZ-10 (Supplementary Material). Although the number of the tested strains is not sufficient to make the conclusions the given results could indicate, the host selected for the propagation of the phage may have an impact on the host range of the virus. The stability in the solution is another important feature when the phages are considered for therapeutic application. One of the primary properties to be measured is the thermal stability and the withstanding ability of the phages to survive in the acidifying environment. It has been well documented that the phages sensitive to low pH in vitro might be completely inactivated by the gastric acid, bile salts, or even acidic food contents in the intestine during the oral application of phages [45,46]. The UZM3 suspension appears to be stable at the storage temperature (4 °C) as well as at body temperature (37 °C) for at least 6 h (observation time). At the same time, it can withstand a wide range of acidity (pH 3–11) from four to six hours. It is known that pH values in the different parts of the body vary from pH 2.0 (gastric acid) to pH 8.0 (urine). Although we observed that the concentration had decreased by one to two logarithmic units within this time period, the endpoint number of phage particles (10^6^) during the first 2 h of the experiment might be still acceptable to reach the therapeutic effect, especially when administered in higher concentrations [47].

Based on the one-step growth curve, the burst size of the phage UZM3 is only about 30 virions per cell produced after each infectious cycle. That may not seem high enough for the beneficial outcome of the phage application in therapy, but the short latent period of 16 min may compensate for the burst size that is comparatively small compared to other phages used for the therapy [47]. In addition, the gene for endolysin detected in the genome of UZM3 is produced during the replication of phage and leading to the lysis of the host cell is of protein nature. Consequently, the activity of the produced lysine can be affected by the changes in temperature or acidity leading to the ineffective application of the phage [47].

So far, many mechanisms have been identified through which the bacterium protects itself from viral invasion, among which the following are best characterized: modification of cell surface molecules to prevent phage adsorption, CRISPR/Cas systems, and activation of important metabolic processes, which consequently limit virus replication [48]. However, phages also have different ways to effectively evade the host’s “immune response”, namely, DNA modification, methylation, RNA repair mechanisms and synthesis of own transport RNAs to prevent host attacks on the translation machinery, synthesis of anti-CRISPR/Cas proteins, and others [48]. In the case of phage UZM3, the existence of the methyltransferase genes (Figure 5) may indicate that the phage has the tendency to protect itself, while inside the cell, from the genome restriction mechanisms of bacteria. Methyltranferase actively methylates the genome of the phage to avoid its degradation by host-derived endonucleases [49].

The first clinical application of UZM3 had a negative outcome, which can be attributed to coexisting debilitating conditions (cancer, COVID 19) or other non-phage factors, the analysis of which is beyond the scopes of our paper. Nevertheless, based on its genomic and biological characterization, we can consider UZM3 to be used for treating antibiotic-resistant forms of *B. fragilis* infections. For the better evaluation of the therapeutic properties, further studies on the safety and efficacy of the phage should be performed.

## 5. Conclusions

The lytic nature of the phage, absence of virulence genes, and other encoded proteins discussed above make UZM3 an acceptable agent for its use in phage therapy. In particular, phage UZM3 may be used for the treatment of *B. fragilis*-induced diarrhea, wound and septic infections, and potentially can be considered for prophylaxis of colon cancer which is believed to be associated with enterotoxigenic strains of *B. fragilis.* High stability at various temperature and in pH environments, as well as short latent period, is another property of the UZM3 that gives hope that the phage will withstand the in vivo challenges during its clinical application. Nevertheless, factors such as geographical restriction of the spectrum of activity, the behavior of the phage in the presence of other bacterial viruses (in a cocktail), or route of administration should be evaluated and taken into account when considering the phage for therapeutic treatment.

## Supplementary Materials

The following supporting information can be downloaded at: https://www.mdpi.com/article/10.3390/v15051042/s1, Table S1: Results of the host range assay title; Table S2: Annotation table.
