# Supplementary material for: Characterization of a Bacteriophage GEC_vB_Bfr_UZM3 Active against Bacteroides fragilis
Source: Viruses. 2023 Apr 25;15(5):1042. doi: 10.3390/v15051042 (PMC10222676; doi:10.3390/v15051042)
Supplement: Supplementary file 1 [file viruses-15-01042-s001.zip › Supplementary material 1. Bacterial strains.pdf]

B.fragilis isolates

	Name	Origin	VA7			MTK			UZM3/ A7			UZM3 /UZ-10		
		Titer	10^8	10^7	10^6	10^8	10^7	10^6	10^8	10^7	10^6	10 ^8	10^7	10^6
1	A5	Fecal sample (Georgia)	1	R	R	2	1	R	3	2	1	R	R	R
2	A6	Fecal sample (Georgia)	4	4	4	3	2	1	3	2	1	3	2	1
3	A7	Fecal sample (Georgia)	3	2	1	3	2	1	3	2	1	3	2	1
4	E6	Fecal sample (Georgia)	4	4	4	3	2	1	3	2	1	R	R	R
5	33	Peritonitis (Belgium)	3	2	1	3	2	1	3	2	1	R	R	R
6	S11	Fecal sample (Georgia)	4	4	4	4	4	4	4	4	4	2	R	R
7	UZ-10	Osteomyelitis drainage(Belgium)	R	R	R	R	R	R	R	R	R	4	3	2
8	S18	Fecal sample (Georgia)	R	R	R	R	R	R	R	R	R	R	R	R
9	E8	Fecal sample (Georgia)	R	R	R	R	R	R	R	R	R	R	R	R
10	S16	Fecal sample (Georgia)	1	R	R	2	1	R	1	1	R	R	R	R
11	E1	Fecal sample (Georgia)	R	R	R	1	R	R	R	R	R	R	R	R
12	S10	Fecal sample (Georgia)	R	R	R	R	R	R	R	R	R	3	2	1
13	E3	Fecal sample (Georgia)	2	R	R	2	R	R	2	1	R	3	2	1
14	S1	Fecal sample (Georgia)	R	R	R	R	R	R	R	R	R	R	R	R
15	S2	Fecal sample (Georgia)	4	3	3	4	4	4	4	4	3	R	R	R
